# Multiorgan tropism of SARS-CoV-2 lineage B.1.1.7

**DOI:** 10.1007/s00414-021-02691-z

**Published:** 2021-09-06

**Authors:** Benjamin Ondruschka, Fabian Heinrich, Maja Lindenmeyer, Carolin Edler, Dustin Möbius, Jan Czogalla, Axel Heinemann, Fabian Braun, Martin Aepfelbacher, Marc Lütgehetmann, Tobias B. Huber

**Affiliations:** 1 Institute of Legal Medicine, University Medical Center Hamburg-Eppendorf, Hamburg, Germany; 2 III. Department of Medicine, University Medical Center Hamburg-Eppendorf, Hamburg, Germany; 3 Institute of Medical Microbiology, Virology and Hygiene, University Medical Center Hamburg-Eppendorf, Hamburg, Germany

**Keywords:** Autopsy, SARS-CoV-2, Variants of concern, Organ tropism, B.1.1.7

## Abstract

Due to the development of novel functionalities, distinct SARS-CoV-2 variants such as B.1.1.7 fuel the current pandemic. B.1.1.7 is not only more transmissible, but may also cause an increased mortality compared to previous SARS-CoV-2 variants. Human tissue analysis of the SARS-CoV-2 lineage B.1.1.7 is urgently needed, and we here present autopsy data from 7 consecutive SARS-CoV-2 B.1.1.7 cases. The initial RT-qPCR analyses from nasopharyngeal swabs taken post mortem included typing assays for B.1.1.7. We quantitated SARS-CoV-2 B.1.1.7 viral load in autopsy tissue of multiple organs. Highest levels of SARS-CoV-2 B.1.1.7 copies normalized to ß-globin were detected in the respiratory system (lung and pharynx), followed by the liver and heart. Importantly, SARS-CoV-2 lineage B.1.1.7 was found in 100% of cases in the lungs and in 85.7% in pharynx tissue. Detection also in the kidney and brain highlighting a pronounced organ tropism. Comparison of the given results to a former cohort of SARS-CoV-2 deaths during the first wave in spring 2020 showed resembling organ tropism. Our results indicate that also SARS-CoV-2 B.1.1.7 has a relevant organ tropism beyond the respiratory tract. We speculate that B.1.1.7 spike protein’s affinity to human ACE2 facilitates transmission, organ tropism, and ultimately morbidity and mortality. Further studies and larger cohorts are obligatory to proof this link.

Due to the development of novel functionalities, distinct SARS-CoV-2 variants such as B.1.1.7 fuel the current pandemic. SARS-CoV-2 lineage B.1.1.7 was first identified in the UK and spread in multiple regions worldwide [1]. B.1.1.7 is not only more transmissible, but may also cause an increased mortality compared to previous SARS-CoV-2 variants [2]. We have previously reported a multiorgan tropism of the initial SARS-CoV-2 lineage [3, 4] that can also associate with organ outcome [5]. Organ tropism thereby correlated with the presence of comorbidities including chronic kidney disease and diabetes [5]. The first case report on autopsy results of a B.1.1.7 fatality was published recently in Int J Legal Med [6].

Human tissue analysis of the SARS-CoV-2 lineage B.1.1.7 is urgently needed, and we here present autopsy data from 7 consecutive SARS-CoV-2 B.1.1.7 cases (clinical data in Table 1). These individuals died out-of-hospital (*n* = 3) and in-hospital (*n* = 4). The cohort was of high age (median 75 years, interquartile range 52–78 years) with a sex ratio of 4:3 (male:female). The initial RT-qPCR analyses from nasopharyngeal swabs taken post mortem included typing assays for B.1.1.7 in the form of N501Y and del HV69//70 as recently established [7] in combination with screening for E484K and P681H (TIB Molbiol, Berlin, Germany). All out-of-hospital deceased were first tested positive post mortem. Full autopsies were done in all cases following guidelines on the handling of COVID-19 deaths [8]. Organ samples were processed and analyzed for the E gene of SARS-CoV-2.

**Table 1:** Summary of case characteristics including sex, age, post-mortem interval (PMI), number of pre-existing medical conditions and places of death (*OH* out of hospital, *NW* normal ward, *ICU* intensive care unit). Forensic aspects of no. 1 have been published as case report [6]

No	Sex	Age	PMI	Place of death	Comorbidities (*n*)	Airway (*n*)	Cardiovascular (*n*)	Kidney (*n*)	Brain (*n*)	Metabolism (*n*)	Other (*n*)
1	f	70	6	OH	4	1	2	1	0	0	0
2	m	76	2	NW	4	0	3	0	0	0	0
3	f	78	6	OH	4	1	2	0	0	1	0
4	m	85	4	ICU	4	0	3	0	0	0	1
5	m	45	2	ICU	2	0	1	0	0	0	1
6	m	75	12	ICU	5	1	2	1	0	1	0
7	f	52	8	OH	3	0	2	0	0	1	0

We quantitated SARS-CoV-2 B.1.1.7 viral load in autopsy tissue of multiple organs. Highest levels of SARS-CoV-2 B.1.1.7 copies normalized to ß-globin were detected in the respiratory system (lung and pharynx), followed by the liver and heart (Fig. 1). Importantly, SARS-CoV-2 lineage B.1.1.7 was found in 100% of cases in the lungs (7/7), in 85.7% in pharynx tissue (6/7), in 57.1% in the liver and heart (4/7 each), and in 42.9% in the kidney and brain (3/7) highlighting a pronounced organ tropism (Fig. 1). Comparison of the given results to a former cohort of 27 SARS-CoV-2 deaths during the first wave in spring 2020 [3] showed resembling organ tropism (Fig. 1).

**Fig. 1:**
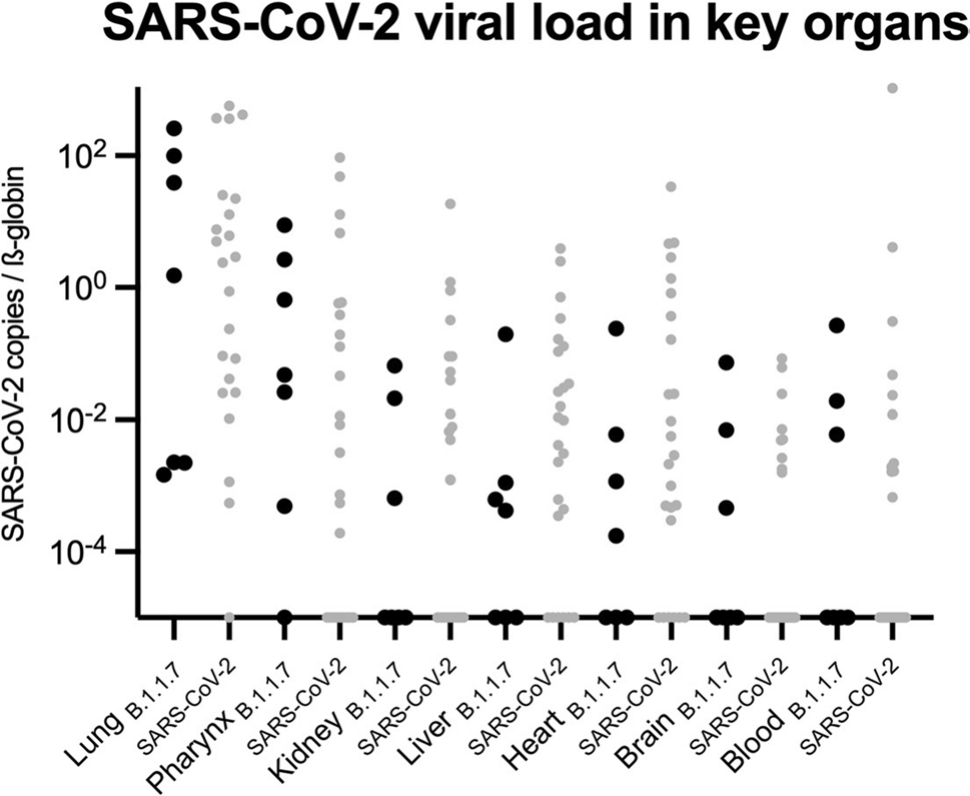
Multi-organ tropism of the SARS-CoV-2 B.1.1.7 lineage. SARS-CoV-2 viral load in key organs, documenting organotropism of B.1.1.7 (virus copies normalized to ß-globin). For comparative reasons, values from Puelles et al. [3] were opposed in grey dots as SARS-CoV-2 original lineage values and were also normalized to ß-globin

Our results indicate that also SARS-CoV-2 B.1.1.7 has a relevant organ tropism beyond the respiratory tract. We speculate that B.1.1.7 spike protein’s affinity to human ACE2 facilitates transmission, organ tropism, and ultimately morbidity and mortality. Further studies and larger cohorts are obligatory to proof this link and our forensic discipline is again one of the key players for this task.
